# Can an experimental white noise task assess psychosis vulnerability in adult healthy controls?

**DOI:** 10.1371/journal.pone.0192373

**Published:** 2018-02-15

**Authors:** Maider Gonzalez de Artaza, Ana Catalan, Virxinia Angosto, Cristina Valverde, Amaia Bilbao, Jim van Os, Miguel Angel Gonzalez-Torres

**Affiliations:** 1 Department of Neuroscience, University of the Basque Country, Leioa, Basque Country, Spain; 2 Department of Psychiatry, Basurto University Hospital, Bilbao, Spain; 3 Research Unit, Basurto University Hospital, Bilbao, Spain; 4 Department of Psychiatry and Psychology, South Mental Health Research and Teaching Network, EURON, Maastricht University Medical Centre, Maastricht, The Netherlands; 5 King´s College London, King´s Health Partners, Department of Psychosis Studies, Institute of Psychiatry, London, United Kingdom; United (Osaka U, Kanazawa U, Hamamatsu U Sch Med, Chiba U and Fukui U) Graduate School of Child Development, JAPAN

## Abstract

**Background:**

This is an extension of a paper published earlier. We investigated the association between the tendency to detect speech illusion in random noise and levels of positive schizotypy in a sample of 185 adult healthy controls.

**Materials and methods:**

Subclinical positive, negative and depressive symptoms were assessed with the Community Assessment of Psychic Experiences (CAPE); positive and negative schizotypy was assessed with the Structured Interview for Schizotypy-Revised (SIS-R).

**Results:**

Speech illusions were associated with positive schizotypy (OR: 4.139, 95% CI: 1.074–15.938; p = 0.039) but not with negative schizotypy (OR: 1.151, 95% CI: 0.183–7.244; p = 0.881). However, the association of positive schizotypy with speech illusions was no longer significant after adjusting for age, sex and WAIS-III (OR: 2.577, 95% CI: 0.620–10.700; p = 0.192). Speech illusions were not associated with self-reported CAPE measures.

**Conclusions:**

The association between schizotypy and the tendency to assign meaning in random noise in healthy controls may be mediated by cognitive ability and not constitute an independent trait.

## Introduction

The presence of psychotic features such as hallucinations, delusions or disorganized thinking is common in a wide range of mental disorders [1].

The prevalence of psychotic experiences in the population is difficult to assess given a range of methodological issues. However, numerous studies in recent years have emerged suggesting that psychotic experiences are more common in the general population than it was thought [2,3,4].

Some individuals without psychiatric history may manifest an attenuated form of hallucinations, of which only a minority will develop a psychotic or other mental disorder over time [5]. Such individuals may be relatives of patients with schizophrenia or individuals with psychometric schizotypy, or participants with physiological, neurological and cognitive characteristics similar to hallucinations found in patients with schizophrenia [6].

Psychotic experiences including auditory verbal hallucinations (AVH) may be phenomenologically and temporally continuous across different levels of clinical severity, ranging from subclinical psychotic experiences in the general population to full-blown psychotic disorder [7,8].

Contemporary models of psychosis suggest that an increase in mesolimbic dopaminergic neurotransmission occurs during the prodromal phase of psychosis, not corresponding to normal learning mechanisms [9]. Dysregulation of dopamine transmission, associated with alterations in top-down processing, causes neutral or irrelevant stimuli—associated with both external and internal representations—to be interpreted incorrectly. The tendency to assign altered meaning or emotional value to a neutral or irrelevant stimulus (aberrant salience) drives the individual to develop a cognitive scheme that alters its ability to process the experience and the surrounding environment appropriately [10,11].

Evidence suggests that alterations in salience attribution mediate the continuum of experiences of subclinical and attenuated psychoses to the sustained expression of psychotic disorder [4].

In order to explore the aberrant salience hypothesis in adults with psychotic disorders, their siblings and general population, a tool was developed to induce speech illusions, the "White Noise Task" (WN). It was first introduced in a study in 2011 [12]. Top-down processing was analyzed by detecting individuals who experienced speech illusions caused by this task and its association with variables of vulnerability to psychosis. The study found that the tendency to detect speech illusions was more frequent in the group of patients with psychotic disorder followed by the group at elevated familial risk. It also revealed that speech illusions were associated in healthy controls with high levels of positive schizotypy.

An attempt at replication was published by Catalan and colleagues, showing replication of the finding that patients had higher rates of WN than controls [13]. However, no association was found between WN and schizotypy in the control group.

In another study, speech illusions were examined in relation to psychotic phenomena in large general population sample of pre-adolescents, using an abbreviated version of the WN task. In this sample, speech illusions were associated with hallucinatory experiences [14].

Here, we present a novel analysis of the expanded non-clinical sample of the adult population presented in the earlier study by Catalan and colleagues [13]. Thus, the original sample was n = 150; the sample in the current analysis was n = 185. The aim was to determine the rate of speech illusions with the WN task and to analyse the association between speech illusions and schizotypal traits. Given the possibility that any association between WN and indicators of psychosis risk may be mediated by known risk cognitive factors, in particular cognitive ability, analyses were adjusted for IQ.

## Method

### Procedure and sample

In order to recruit a representative general population sample to assess population reference values for white noise speech illusion, a control reference group of 185 participants between 17 and 65 years old was selected in Bilbao, Spain, through public advertisement during the period from July 2012 to April 2015.

Inclusion criteria were sufficient knowledge of the Spanish language, intelligence quotient IQ> = 70 according to the Weschsler Adult Intelligence Scale (WAIS-III) and no first-degree relatives with a psychotic disorder, as reported by the participant.

### Ethical issues

The study was approved by the Ethics Committee of Clinical Research of Basurto University Hospital. Participants were given verbal and written detailed explanation about the study and its procedures. Before the start of the first assessment, written informed consent was obtained from all participants. Confidentiality of data was maintained using a unique research ID for each respondent, enabling analysis of individual data without the use of names or other identifiers.

### Instruments

All the interviews and assessments were carried out at Basurto University Hospital by psychologists and psychiatrists, trained in the use of these specific instruments.

#### White noise task [12]

Participants wore earphones and were presented 1 of 3 different types of stimuli: (1) white noise only, (2) white noise + clearly audible neutral speech and (3) white noise + barely audible neutral speech. Stimuli 2 and 3 were not separate conditions; the intermixing of white noise stimuli with audible speech was presented in order to create a higher level of expectancy, thus occasioning levels of top-down processing. Participants were presented 25 fragments of each in random order and asked to respond to each by pressing 1 of 5 buttons hereafter referred to as: 1: positive speech illusion (endorsed hearing positive voice), 2: negative speech illusion (endorsed hearing negative voice), 3: neutral speech illusion (endorsed hearing neutral voice), 4: no speech heard and 5: uncertain; this latter option was included in order to make the ratings of 1–3 more conservative. The recordings were delivered using stimulations software E-prime 1.1 (Psychology Software Tools, Pittsburgh, Pennsylvania) and stimuli were reproduced in random order. The length of the task was approximately 15 min.

The rate of hearing a voice in the white noise-only condition (25 trials) was the variable of interest in the analyses. As white noise speech illusion scores for positive, negative and neutral voices were highly skewed, the 3 outcomes were analysed as dichotomous variables. A variable “any speech illusion” was constructed denoting the presence of at least two instances of any positive, negative or neutral voice perceived in white noise (speech illusion present versus not present), in agreement with previous work [13,14]. In order to examine whether the white noise task was sensitive particularly to affectively salient speech illusions rather than neutral speech illusions, a composite variable was constructed reflecting any positive or negative speech illusions.

#### Wechsler adult intelligence scale-III [15]

General cognitive abilities were assessed for an indication of intellectual functioning (IQ) using the short form of the WAIS-III that includes Information, Block Design, Digit Symbol and Arithmetic subtests.

#### Structured Interview for Schizotypy-Revised (SIS-R) [16]

The SIS-R is a structured interview used to determine a broad range of schizotypal symptoms and signs: the positive, negative and disorganization dimensions of the subclinical psychosis phenotype. Items can be scored on a 4-point scale from absent (score 0) to severe (score 3). Positive schizotypy covers the symptoms referential thinking (2 items), as well as magical ideation, illusions, psychotic symptoms and suspiciousness (total of 6 items). Negative schizotypy contains the signs of social isolation, introversion, restricted affect and poverty of speech (4 items). Mean schizotypy scores for these dimensions were calculated, resulting in a positive schizotypy and negative schizotypy score.

#### Community assessment of psychotic experiences (CAPE) [17]

This self-report questionnaire rates attenuated affective and non-affective psychotic experiences. The CAPE measures, on a dimensional scale, frequency of, as well as distress associated with, these subclinical psychotic experiences. The frequency score is measured on a 4-point scale from: 1 = never to 4 = nearly always. The degree of distress associated with the subclinical psychotic experience is also measured. The CAPE includes dimensions of positive (20 items), negative (14 items) and depressive (8 items) symptoms associated with the subclinical psychosis phenotype in the general population on a 4-point scale with labels ranging from1 = not distressed to 4 = nearly always. For the current analyses, mean scores of frequency of positive and negative symptoms were used.

### Analyses

To study the association between SIS-R scales and CAPE scales on the one hand, and white noise on the other, logistic regression was used, with “any speech illusion” as dependent variable and SIS-R or CAPE scales as independent variables. Logistic regression models were also performed adjusting for age, sex and IQ. Associations derived from logistic models were expressed as odds ratio (OR) and their 95% confidence interval (CI).

Associations were considered significant at p<0.05, double sided.

Statistical analyses were performed using the STATA software programme, version 13 [18].

## Results

### Sample

Controls were 55.14% males and more than half were single (58.38%). Mean age of controls was 31.81 years old (SD = 11.56). The majority was from middle social class (76.76%) and had had full-time education (38.92%). The majority (92.97%) did not live alone, living either with their parents or with their own family (wife/husband and children). The majority (77.84%) had an occupation in the form of either a job (52.43%) or studies (25.41%). Mean WAIS-IQ was 109.01 (SD = 14.93) (Table 1).

**Table 1:** Socio-demographic and cognition variables https://figshare.com/s/a9b7606c8ef68f9253fd.

		Healthy Subjects (N = 185)
		n (%)
Sex	Male	102 (55.14%)
	Female	83 (44.86%)
Age (years), mean (SD)		31.81 (11.56)
Socio-economic level	Low middle class	13 (7.03%)
	Middle class	142 (76.76%)
	High middle class	30 (16.22%)
Residence	Parents	89 (48.11%)
	Partner/Children	83 (44.86%)
	Alone	13 (7.03%)
Education	Primary school	3 (1.62%)
	Secondary school	10 (5.41%)
	High school	36 (19.46%)
	Professional training	44 (23.78%)
	Certificate	20 (10.81%)
	Degree/Master	72 (38.92%)
Work status	Unemployed	36 (19.46%)
	Employed	97 (52.43%)
	Student	47 (25.41%)
	Retired	2 (1.08%)
	Others	3 (1.62%)
Marital status	Single	108 (58.38%)
	Married/Partner	73 (39.46%)
	Separated/Divorced	4 (2.16%)
WAIS-III, mean (SD)		109.01 (14.93)

Data are given as proportions unless otherwise stated.

SD, Standard deviation; WAIS-III, Wechsler Adult Intelligence Scale—Third Edition.

Clinical variables were as follows: mean positive schizotypy score was 0.26 (SD = 0.28) and mean negative schizotypy 0.11 (SD = 0.23); CAPE positive dimension 0.22 (0.14) and negative dimension 0.50 (SD = 0.30). 12.97% of controls perceived at least two instances of any positive, negative or neutral voice in white noise (Table 2).

**Table 2:** SIS-R and CAPE scores in healthy participants https://figshare.com/s/0b71b7caee297f3f2050.

		Mean (SD)
**SIS-R**	Positive schizotypy	0.26 (0.28)
	Negative schizotypy	0.11 (0.23)
**CAPE**	Positive dimension	0.22 (0.14)
	Negative dimension	0.50 (0.30)
**Speech illusion** **n (%)**	Yes	24 (12.97)
	No	161 (87.02)

SD = standard deviation; SIS-R, Structured Interview for Schizotypy-Revised; CAPE, Community Assessment of Psychic Experiences (frequency scores).

### Schizotypy and speech illusions

Any speech illusion was associated with positive schizotypy (OR: 4.139, 95% CI: 1.074–15.938; p = 0.039) but not with negative schizotypy (OR: 1.151, 95% CI: 0.183–7.244; p = 0.881). However, the association of positive schizotypy with speech illusions disappeared after adjustment for age, sex and WAIS-III (OR: 2.577, 95% CI: 0.620–10.700; p = 0.192). Speech illusions were not associated with the CAPE positive (OR: 7.221, 95% CI: 0.471–110.497; p = 0.155) and negative scales (OR: 1.250, 95% CI: 0.294–5.315; p = 0.762) (Table 3).

**Table 3:** Associations between white noise speech illusion and SIS-R and CAPE https://figshare.com/s/09c048d70d0c4ef3ed0c.

	Any speech illusion			
	*Not adjusted*		Adjusted*	
	*OR (95% CI)*	*p*	*OR (95% CI)*	*p*
**SIS-R**				
Positive schizotypy	4.139 (1.074–15.938)	0.039	2.577 (0.620–10.700)	0.192
Negative schizotypy	1.151 (0.183–7.244)	0.881	1.266 (0.164–9.731)	0.820
**CAPE**				
Positive dimension	7.221 (0.471–110.497)	0.155	9.672 (0.561–166.683)	0.118
Negative dimension	1.250 (0.294–5.315)	0.762	2.182 (0.448–10.626)	0.334

OR, odds ratio; CI, confidence interval; SIS-R, Structured Interview for Schizotypy-Revised; CAPE, Community Assessment of Psychic Experiences (frequency scores).

*Adjusted for age, sex and CI (Wechsler Adult Intelligence Scale).

## Discussion

The degree to which WN reflects vulnerability for expression of psychosis in healthy participants, possible reflecting alterations in processing top-down or aberrant salience in healthy population, remains uncertain. No associations were apparent with self-reported measures of psychotic experiences. While WN was associated with interview-based measures of positive schizotypy, this appeared to be mediated to a large extent by other variables including cognitive ability.

In accordance with the current findings, a recent research [19] speculates that neurocognitive mechanism underlying perceptual abnormalities might differ between psychotic patients and the non clinical population, based on the findings showing no association between white noise speech illusion and subtle psychosis expression in a large general population adolescent and young adult twin cohort (n = 704).

The strengths of our study were, first, the use of both the SIS-R and CAPE scales, designed to measure the prevalence of positive experiences in the general population. Second, we included adequate control for cognitive ability. Lastly, the use of a representative sample in terms of age and education, reducing the risk of bias. A potential weakness is that an even larger sample may be required to detect the small association that may remain after adjustment for confounders. Similarly, sensitivity of the analyses may be enhanced if preferentially young people are included, given the high prevalence of psychotic experience around adolescents. This latter factor may explain the finding of a positive association between WN and hallucinatory experiences in an earlier study [14].

In conclusion, although there was an apparent association between the tendency to detect speech illusions in random noise and interview-based positive schizotypal traits, the association appeared to be mediated to a large extent by cognitive ability. Research in larger samples and/or uniformly young people may shed more light on this issue.
